# The Correlation Between Screen Time and the Probability of Developing Autism Spectrum Disorder

**DOI:** 10.7759/cureus.73231

**Published:** 2024-11-07

**Authors:** Sherzad Mosa, Farhad Armishty, Marwa Haji, Media Ali, Parween Ahmed, Snor Husain, Khalida Suleiman

**Affiliations:** 1 College of Medicine, University of Zakho, Zakho, IRQ

**Keywords:** asd, autism, children, electronic devices, screen time

## Abstract

Background

Autism spectrum disorders (ASDs) are a collection of neurological, psychological, and developmental anomalies that manifest in early life, affecting individuals across all racial, cultural, and socioeconomic groups. Its prevalence has grown significantly over the past 20 years. Exposure to digital devices has increased alongside the rise in ASD prevalence. Research suggests that prolonged screen time can negatively impact a child's brain development, language, literacy, and cognitive function. The aim of this study was to investigate the correlation between screen time and the probability of developing autism spectrum disorder.

Methodology

This study employed a case-control design to examine 231 children in Zakho City diagnosed with an autism spectrum disorder. The study was conducted from October 1, 2023, to March 1, 2024. The participants included neurotypical individuals and individuals with autism. Data were gathered through standardized questionnaires and analyzed using IBM SPSS Statistics for Windows, Version 26 (Released 2019; IBM Corp., Armonk, New York). The study was approved by the College of Medicine/University of Zakho, Kurdistan Region, Iraq, and ethical permission was obtained.

Results

The study revealed a uniform age distribution between cases and controls, with a majority of male participants and a smaller percentage of female participants. ASD patients had a significantly longer duration of exposure to electronic devices compared to controls, with cases averaging 3.61 hours of screen time daily (t-test: t = 0.0001).

Conclusion

In summary, screens have a major impact on children's neurodevelopment and may increase their risk of developing ASD. However, no appreciable distinction was observed between children diagnosed with ASD and those without regarding early exposure to screens. Our findings can be used to create guidelines for children's media consumption and to raise awareness of this issue. Further research is needed to evaluate the association.

## Introduction

In 1943, Leo Kanner originally employed the term "autism" to characterize a distinct syndrome observed in young children. It pertains to autism spectrum disorders (ASDs), a collection of neurological, psychological, and developmental anomalies that manifest in early life [1-4].

ASD patients often exhibit nonverbal communication and a lack of social behavior. More particularly, they avoid making eye contact, struggle in social situations, and engage in repetitive activities and body language. Due to their social isolation and lack of confidence, which can lead to depression and anxiety, people with autism generally have a lower quality of life [5, 6].

Every race, culture, and socioeconomic group is affected by ASD. However, diagnosis rates vary greatly across different populations [3, 7]. Though there is a 3:1 to 4:1 male preponderance, females are more likely than males to have an intellectual disability [3, 8, 9].

As of now, the CDC (Centers for Disease Control and Prevention) believes that one child out of every 59 has ASD. While 39% of children have their initial evaluation after turning four, most receive their diagnosis at three years old [10, 11].

Over the past 20 years, the prevalence of ASD has seen the greatest increase among exceptional children's groups [12]. Research suggests that autism may be caused by a combination of genetic and environmental factors, even though a single cause is not yet known [6]. It is noteworthy that early exposure to digital devices such as cell phones, tablets, and televisions has recently increased alongside the rise in ASD prevalence [12].

According to a 2019 World Health Organization report, infants under the age of one should not be exposed to screens. The recommendations also include guidelines for children under five, emphasizing good sleep patterns, limited sedentary behavior, and safe physical activity [13]. The American Academy of Pediatrics recommends no more than one hour of screen time during the week and three hours on weekends for children aged two to five. It also advises against unsupervised screen time for children younger than two [14].

Numerous studies conducted in various nations indicate that young children who spend more time on screens may experience negative health effects, such as mood fluctuations, delayed language development, and traits similar to autism, including hyperactivity, a brief attention span, and impatience. Furthermore, a child who spends excessive time on screens often interacts poorly with their parents. This impacts child development negatively, as interactions between parents and children are beneficial for a child's language development, particularly for word learning and retention [15].

Children are spending more time in front of screens than ever before. Children now watch screens for three to six hours per day, making it their most prolonged daily activity besides sleeping, due to the increased availability of home media worldwide. According to this research, a significant number of children under the age of two spend around one-third of their waking hours watching electronic screens [8].

While the benefits of electronics are undeniable, prolonged screen usage negatively impacts a child's brain development. Research conducted in 2019 examined the potential effects of increased screen usage on the white matter development of toddlers and preschoolers. White matter controls language, cognitive function, and literacy [6].

Social interactions activate a brain process that strengthens one's perception of their bond with others. Early language learning is primarily influenced by the linguistic environment, which directly stems from social contact [8].

Thus, the purpose of this study was to investigate the correlation between screen time and the probability of developing autism spectrum disorder.

## Materials and methods

Study design

A case-control design involving face-to-face interviews was utilized, with controls comprising normal individuals and cases comprising autistic individuals.

Selection of subjects

The study, conducted from October 2023 to March 2024, involved participants from various centers who were invited to fill out a questionnaire. About 231 children attend autism centers in Zakho City, Kurdistan Region, Iraq, consisting of 180 males and 51 females. The study included 199 control volunteers, with 103 males and 96 females.

The criteria of the study

Inclusion criteria included any children in autism centers diagnosed with autism with any type of autism at an age between 2 and 12 years. The study did not involve any children with cerebral palsy, mental retardation, difficulty hearing, or Down syndrome.

The patient information

The study involved two groups of children: the control group comprised children from Zakho kindergartens and children who visited the Zakho General Hospital in Zakho City for medical care, while the case group consisted of children from Zakho autism centers. Information was collected using a standardized questionnaire. This included details on the mother's health during pregnancy, the baby's health following delivery, the type and duration of feeding, and sociodemographic characteristics. Data on screen exposure were also gathered for both the case and control groups.

Definition of variable

Autism is classified as a developmental disease by both the World Health Organization's ICD-10 (International Classification of Diseases, Tenth Revision) and the American Psychiatric Association's DSM-5 (Diagnostic and Statistical Manual of Mental Disorders, Fifth Edition). Autism spectrum disorder (ASD) is the recognized term for autism [1]. 

Ethical approval

The College of Medicine/University of Zakho, Kurdistan Region, Iraq, ethics committee authorized the study proposal. This approval is documented by a letter issued with the reference number (AUG23/E10) on August 6, 2023. Prior to sample collection, parents of the children were contacted for permission to participate in the study, and informed written agreements were obtained from all participants.

Statistical analysis

The data were analyzed using IBM SPSS Statistics for Windows, Version 26 (Released 2019; IBM Corp., Armonk, New York) and presented as percentages and numbers. The variables were associated using the t-test and the Pearson chi-square test. For independent variables, logistic regression analysis was employed to determine their true impact on the outcome. Statistical significance was considered for all analyses when the P-value was less than 0.05.

## Results

Males constitute the majority of the cases (77.9%), while females make up a smaller percentage (22.1%). In the control group, the distribution of males (51.8%) and females (48.2%) is more balanced. The average age of cases is 7.84 years, compared to 7.36 years for the controls. The combined mean age of the two groups is 7.66 years, indicating a generally uniform age distribution. As shown in Table 1, the majority of cases (94.8%) and controls (91%) are Muslims and belong to the Kurdish ethnic group.

**Table 1:** Sociodemographic characteristics

Variables		Case, n (%)	Control, n (%)	Total, n (%)
Gender	Male	180 (77.9%)	103 (51.8%)	283 (100%)
	Female	51 (22.1 %)	96 (48.2%)	147 (100%)
Mean age in years (mean ± SD)		7.84 ± 3.04	7.36 ± 2.89	7.66 ± 2.99
Ethnicity	Kurd	219 (94.8%)	181(91%)	400 (100%)
	Arab	12 (5.2)	18 (9%)	30 (100%)
Religion	Muslim	210 (90.9%)	163(81.9%)	273 (100%)
	Yazidi	9(3.9%)	24(12.1%)	33 (100%)
	Christian	12(5.2%)	12(6%)	24 (100%)
Residence	Urban	208 (54.59%)	173 (45.41%)	381 (100%)
	Rural	9 (36%)	16 (64%)	25 (100%)
	Camps	14 (58.3%)	10 (41.7%)	24 (100 %)

The average age of individuals with ASD is 7.84 years, while controls have an average age of 7.36 years (t = 0.296); the mean age difference between the patients and controls is not statistically significant. The mean age at which the cases and controls began using electronic devices is 16.48 months for the cases and 15.45 months for the controls; the t-value of 0.131 indicates no statistically significant difference between the two groups. Compared to controls, who use electronic devices for an average of 2.99 hours per day, cases use them for an average of 3.61 hours. As shown in Table 2, there is a statistically significant difference in the hours of electronic device use between the patients and controls (t = 0.0001).

**Table 2:** The information on various variables comparing cases (individuals with autism spectrum disorder, ASD) and controls (presumably individuals without ASD) *  t-test ** P-value

Variants	Case (mean ± SD)	Control (mean ± SD)	Total (mean ± SD)	t-test
Age, in years	7.84 ± 3.04	7.36 ± 2.89	7.66 ± 2.99	0.296*
Age of diagnosis of ASD, in years	2.91 ± 1.15	-	2.91 ± 1.15	-
Age at which one began using electrical devices, in months	16.48 ± 13.9	15.45 ± 14.24	16.03 ± 14.04	0.131*
Only child, n (%)	53 (60.9%)	34 (39.1%)	87 (100%)	0.132**
Hours of watching electronic devices/day	3.61 ± 2.39	2.99 ± 1.87	3.34 ± 2.20	0.0001*

Table 3 shows no significant difference in the mother's education levels between cases and controls; there is a significant difference in the father's education levels.

**Table 3:** Parent’s education level concerning autism The P-value is calculated by chi-square. A P-value less than 0.05 is considered statistically significant. The chi-square value for the mother's education level is 0.846 and the father's education level is 66.107.

Education level	Mother's education level		P-value	Father's education level		P-value
	Case, n (%)	Control, n (%)		Case, n (%)	Control, n (%)	
Primary school and below	99 (42.9%)	88 (44.2 %)	0.839	93 (40.3%)	60 (30.2%)	0.045
Middle school	57 (24.7%)	46 (23.1%)		72 (31.2%)	59 (29.6%)	
College degree	62 (26.8%)	57 (28.6%)		50 (21.6%)	65 (32.7%)	
Advance degree	13 (5.6%)	8 (4%)		16 (6.9%)	15 (7.5%)	
Total	231 (100%)	199 (100%)		231(100%)	199 (100%)	

Among the cases, the majority began using electronic devices before the age of one year (143 individuals), followed by those who started between one and two years (50 individuals), and those over two years (35 individuals). Among the controls, a similar distribution is observed, with most starting to use electronic devices before the age of one year (113 individuals), followed by those who began between one and two years (31 individuals), and those over two years (31 individuals). The P-value for comparing the age of first exposure to electronic devices between cases and controls is 0.531, indicating no significant difference between the two groups. Table 4 summarizes the data on first exposure to electronic screen devices.

**Table 4:** Individuals in the study sample were first exposed to electronic screen devices The P-value is calculated by chi-square. A P-value less than 0.05 is considered statistically significant. The chi-square value is 1.267.

Age of watching devices	Case, n (%)	Control, n (%)	P-value
<1 year	143 (62.72%)	113 (64.58%)	0.531
1-2 years	50 (21.93.%)	31 (17.71%)	
>2 years	35 (15.35%)	31 (17.71%)	
Total	228 (56.58%)	175 (43.42%)	

**Figure 1:**
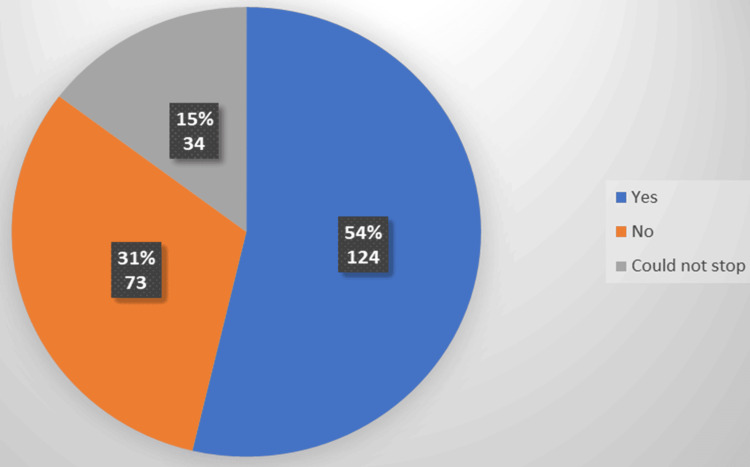
Improvement after discontinuing electronic screen exposure

Figure 1 illustrates how the children have improved while giving up screen time; most (53.7%) had excellent responses. However, a small portion of them (14.7%) were addicted to screen time. Furthermore, 31.6% did not improve. 

## Discussion

Our research was identified as the inaugural study in Kurdistan investigating the correlation between early and prolonged exposure of children to electronic screen devices and the onset of autism spectrum disorder, following an online search for related studies on this significant topic.

There was a 3.5:1 male-to-female ratio (P = 0.0001), which was somewhat lower than the 4.3:1 ratio reported by the CDC for the United States in 2016. The CDC stated that the range for this size difference in the United States was between 3.4:1 and 4.7:1 [16].

In this study, the mean duration of electronic device use among ASD patients was 3.61 hours, with a noteworthy P-value of 0.001, compared to 2.99 hours in the control group.

Additionally, certain studies have indicated a correlation between extended screen usage across different devices and an increased risk of developmental disorders, including autism spectrum disorder. Alrahili et al. found a significant (P < 0.05) correlation between screen time and elevated scores on social communication questionnaires, illustrating a link between the frequency of ASD symptoms and longer screen time [17]. Device exposure was also associated with a greater likelihood of autism-like behaviors, as shown by Chen et al.'s comparison of children who have and have not used screens (AOR = 1.61, 95% CI 1.18-2.21) [18].

According to Bibi et al., children with autism spectrum disorder who spent two or more hours in front of screens displayed irregularities in responsiveness, with 48% exhibiting slower reactions in everyday situations and 19% not responding at all [19]. Wu et al. reported that children with higher screen time scored significantly higher on the strengths and problems questionnaire (P < 0.05) [20]. Dehiol et al. found that individuals with ASD spent over four hours per day (P = 0.001) watching different screens, primarily televisions [8]. Research by Md Zaki Fadzil et al. indicated that children who scored a mean of three on the 20-item M-CHAT-R parent-report screening measure were more likely to develop ASD if they spent more than three hours in front of a screen [21].

However, our findings were contradicted by other studies. For instance, Hill et al. did not find a statistically significant difference between the ASD group and other control groups with respect to screen exposure [22]. Melchior et al. reported that while screen time is associated with an intermediate risk of neurodevelopmental problems, it is not linked to a high risk [23].

More than 84.65% of patients with ASD began using electronic screens within the past two years (62.72% at <1 year and 21.93% at age 1-2 years), compared to 82.29% of participants in the control group who began using screens within the past two years, with a P-value of 0.531, which is not statistically significant.

Our findings are supported by Md Zaki Fadzil et al.'s research [21], indicating no significant correlation between the duration of exposure and symptoms of ASD (P = 0.432). However, Dehiol et al. discovered a strong correlation (P = 0.001) between early screen exposure and ASD development [8]. Chen et al. found that children exposed within the first year of life had a significantly greater risk of ASD compared to other controls (AOR = 2.13, 95% CI 1.54-2.94) [18].

There was a notable improvement in these children's symptoms once screen use was reduced, a finding supported by Dehiol et al. [8]. We conclude that, rather than allowing children to watch television or use smartphones, parents should explore other ways to engage them.

Limitations 

The limitation of this study is the smaller sample size, which restricts the generalizability of the results to the broader population. However, as the first study conducted in Zakho City on this topic, it underscores the need for more multicentric studies with larger sample sizes to validate and align with our findings.

Recommendations 

A larger study is needed in the Kurdistan Region to gather more information and produce more precise results about autism and its associated factors within the region's entire autism population.

Regional seminars should be organized to educate parents about autism and its risk factors. Prominent factors associated with autism, such as screen time, should be explained more explicitly to parents when providing the AAP guidelines for screen use. The amount of time parents allow their children to spend on screens should be either eliminated or severely limited.

## Conclusions

In summary, screens have a major impact on children's neurodevelopment and can significantly increase the risk of developing ASD. However, no appreciable distinction was observed between children diagnosed with ASD and those who did not have early exposure to screens. Our findings can be used to develop guidelines for children's media consumption and to raise awareness of this issue. Further research is needed to evaluate this association.
